# Team-based learning improves knowledge and retention in an emergency medicine clerkship

**DOI:** 10.1186/s12245-019-0222-2

**Published:** 2019-02-11

**Authors:** Arif Alper Cevik, Margaret ElZubeir, Fikri M. Abu-Zidan, Sami Shaban

**Affiliations:** 1 Departments of Internal Medicine, College of Medicine and Health Sciences, United Arab Emirates University, Al Ain, United Arab Emirates; 2 Department of Emergency Medicine, Tawam-John Hopkins Hospital, Al Ain, United Arab Emirates; 3 Department of Medical Education, College of Medicine and Health Sciences, United Arab Emirates University, 17666, Al Ain, United Arab Emirates; 4 Department of Surgery, College of Medicine and Health Sciences, United Arab Emirates University, Al Ain, United Arab Emirates

**Keywords:** Active learning, Team-based learning, Emergency medicine clerkship, Long-term retention, Student perceptions

## Abstract

**Background:**

Team-based learning (TBL) as an instructional pedagogy is increasingly recognized to improve student engagement, value of teamwork, and performance on standardized assessments when compared to traditional lecture-based instruction. The aim of this study is to compare two educational modalities (TBL and didactic/case discussion) on knowledge-based outcome and student perceptions.

**Methods:**

Two emergency medicine clerkship academic years were studied. In the first year, all topics were delivered via didactic presentations along with case discussions. In the second year, eight topics were delivered using TBL while three topics were delivered via didactic/case discussions. Final exam marks were compared. Student satisfaction survey was also conducted and analyzed.

**Results:**

After adjusting for student past performance and exam difficulty, student marks improved in the second year for both TBL and didactic/case discussion topics. The average mark for topics taught via TBL in the second year was significantly higher than the average mark on the same topics taught didactically in the first year by 7.5% (*T* test, *p* < 0.001). The marks for topics taught via TBL showed better improvement comparing to topics taught via didactic/case discussion by 2.3% (ANOVA-RM, *p* = 0.042). Student marks related to TBL topics were significantly higher on the medical exit exam (paired *t* test, *p* = 0.007). Student response to TBL survey was positive.

**Conclusions:**

TBL as part of a blended learning environment facilitated improved knowledge-based performance in an emergency medicine clerkship following end clerkship and medical school exit assessments, suggesting TBL stimulates long-term retention. The high acceptance of TBL among our students suggests a preference of this learning modality to didactic teaching.

## Background

Diverse instructional pedagogies in higher education are being advocated internationally. Team-based learning (TBL) is one such pedagogy introduced relatively recently in several courses across medical curricula [1, 2]. This teacher-directed, interactive, small group, instructional strategy is more structured and less resource intensive [3, 4] than problem-based learning (PBL). Compared with passive, didactic, lecture-based learning, TBL is based on specific constructivist design principles and is more powerful in promoting critical thinking, problem solving, team building, and communication skills that are necessary in medical students’ future clinical practice [5, 6]. TBL is based on techniques that enhance engagement and high-level performance at individual and team levels. These techniques typically include individual preparatory work before class, a test of individual performance on learning from instructor-assigned tasks related to specific outcomes (readiness assurance), followed by a test of team (five to seven students) performance on learning from the same instructor-assigned tasks followed by an application exercise designed to promote application of knowledge, reasoning, problem solving, and team communication skills [2].

Thus, in contrast to didactic lectures, the popularity of TBL has grown in medicine due to its ability to exceed simple coverage of content and foster active discussion in the context of clinical scenarios, thereby ensuring students’ mastery of course content and application of knowledge to solve real-world problems in small groups [5, 6]. Indeed, pedagogies based on constructivist learning theories, such as TBL, have potential for developing advanced lifelong learning skills that have significant practical application in clinical learning environments. Acquisition of these advanced learning skills may culminate in the development of master learners [5] capable of content knowledge retention over extended periods of time.

There are however limited studies comparing TBL to other instructional strategies in the clinical years [7]. Those that exist are varied in their methodology and choice of discipline [8, 9], and others provide conflicting reports regarding knowledge and skills outcomes [10].

The role of the teacher in medical education is multifaceted and includes facilitation, resource development, and curriculum planning [11]. As such, medical educators are often expected to translate contemporary principles of learning into instructional activities and materials that meet student needs and expectations. Thus, similar to diagnosing and managing patient problems, good medical educators diagnose and manage student learning needs. This necessitates judicious application of appropriate learning theories. Indeed, as described by Ertmer and Newby, “learning theories are a source of verified instructional strategies, tactics and techniques” [12].

The instructional emphasis moves from teaching to learning via learner active involvement in the learning process and gradually, by talking to peers, senior students and professors, learners’ abilities to articulate their own understanding of topics. For example, a typical TBL goal in the emergency medicine (EM) clinical clerkship context would not be to teach novice clerkship students’ straight facts about fever but to prepare students to activate prior knowledge about fever and apply relevant facts to a child presenting with fever of unknown cause in the same manner that an EM specialist might. Assessment will be focused on transfer of knowledge and skills in similar cases.

Specific strategies utilized in learning activities underpinned by constructivist principles include situating tasks in real-world contexts, use of modeling and coaching (i.e., cognitive apprenticeship), exploration of multiple perspectives, collaborative learning to develop and share alternative viewpoints, debate, discussion, evidence giving, provision of feedback, reflection, and use of problem solving skills that encourage students to go beyond the information provided [12]. Constructivist theories focus on the active character of the learner, interacting individually and with others resulting in learning being a co-construction and qualitative reorganization of knowledge structures [12]. Students exposed to TBL learning activities develop as learners and gain different competencies that are transferrable.

The College of Medicine and Health Sciences (CMHS), United Arab Emirates University, has been implementing TBL since 2015 in the pre-medical and pre-clinical curriculum while, for the time being, there would be no immediate expectation that clinical clerkship courses would adopt TBL. Nevertheless, cognizant of the pedagogical strengths of TBL, the EM clerkship director adopted TBL in the 2016/2017 EM clerkship. This represented an early adopter TBL in the clinical years at CMHS.

The aim of this study is to compare assessment outcomes and student perceptions following implementation of two educational modalities (TBL and didactic/case discussion) in an EM clerkship. Based on previous findings, we hypothesized that there would be an evident positive impact on student performance following TBL experience in comparison to students who experienced didactic, lecture-based learning on specific EM topics and that students would be receptive to this teaching and learning innovation in the clerkship.

## Methods

### Ethical approval

This study was reviewed and approved by The Research and Graduate Studies Ethics Committee of United Arab Emirates University (ERS-2016-4431).

### Participants

One hundred forty-five final year medical students who trained in the EM clerkship in two consecutive years were included in the study. Seventy-nine of the students were in the 2015/2016 academic year and 66 in the 2016/2017 academic year.

### Study design and setting

This is a retrospective analysis of prospectively collected data of two consecutive academic years in the EM clerkship (2015/2016 and 2016/2017) in the CMHS. While these are two separate cohorts of students, they are very similar in all parameters except for the teaching method. The two groups of students are all Emirati nationals and in their sixth year in the MD Program. They were similarly distributed in gender, age, number of repeating students, and overall performance up to the point of the clerkship (no significant difference in average overall program mark, *t* test, *p* = 0.086, Table 2). Teaching activities were done in the classroom and clinical skills laboratory. Two government-affiliated teaching hospitals are used for clinical teaching and practice for our students. Tawam Hospital, which is affiliated with Johns Hopkins Medicine International, treats approximately 110,000 patients in the emergency department (ED) annually. The ACGME-I accredited Emergency Medicine Residency Program is also located at this hospital. Al Ain Hospital ED treats over 115,000 emergency patients annually. Clinical shifts are located at four different locations of the ED (resuscitation room, urgent care area, fast track area, and a pediatric unit).

### Teaching and assessment in EM clerkship

The EM clerkship is designed based on the curriculum recommendations of the Society for Academic Emergency Medicine and International Federation for Emergency Medicine [13, 14]. The clerkship rotation is 4 weeks long. Depending on curriculum requirements, 11 chief complaint-oriented topics are covered in the clerkship (Table 1).

**Table 1:** EM topics and TBL coverage for 2016/2017 academic year

Topic number	TBL	Topic name
1	√	Abdominal pain
2		Altered mental status
3	√	Cardiac arrest and arrhythmia management
4	√	Chest pain
5	√	Fever in child
6	√	Gastrointestinal bleeding
7		Headache
8	√	Poisoning
9	√	Respiratory distress
10	√	Shock
11		Trauma (multiple)

### Didactic/case discussion process (2015/2016 academic year)

During the 2015/2016 academic year, the topics were presented by the EM clerkship director via didactic/case discussion sessions. Students received 30–45-min presentations followed by case discussions. The presentations included minimum required knowledge related to learning outcomes of the topics. After the didactic day, the students are encouraged to read the topics from previously given resources (hardcopy, softcopy, and online). Students then took weekly multiple choice question (MCQ) exams on topics discussed in the teaching day.

### Team-based learning process (2016/2017 academic year)

During the 2016/2017 academic year, eight topics were selected for TBL by an EM committee including the clerkship director and core faculty members of the EM residency program (Table 1). This was the first time these students were exposed to TBL in their medical curriculum and the first time the EM faculty used TBL as a teaching modality. The clerkship director attended a 2-day professional development course at the end of 2015/2016 academic year. Clerkship rotations include a total of 13 to 18 students. In the TBL sessions, the material was provided to students ahead of time and subsequently they received individual and group readiness assurance tests on that topic. The readiness assurance test includes multiple choice (80%) and true-false (20%) questions. Depending on the content of the topic, 10–20 questions were used in the readiness assurance test. Total time given for readiness assurance test stage was 30–40 min. Self-formed teams of five to six students received feedback on questions answered incorrectly during the team test. Socrative online test application was used for readiness assurance tests. Questions answered incorrectly by any team and additional questions asked by students were further discussed at the end of team test feedback. Appeals from teams, if any, were evaluated and final decision applied to final scores. The application exercises followed, which included three to four interactive case discussions focused on the application of knowledge to real EM scenarios. Readiness assurance process was graded as 60% for individual and 40% for team tests. No grading was applied to the application exercise. Peer-evaluation surveys were not implemented after each TBL. However, a quantitative and qualitative survey regarding TBL was applied at the end of the clerkship.

### Didactic/case discussion process (2016/2017 academic year)

Three topics were delivered using the didactic/case discussion modality. Students were expected to study the topics from the given resources before the class. This is in contrast to the previous year’s method in which students were not expected to study the material before class. However, similar to the previous year, students received short presentations followed by case discussions. The presentations included minimum required knowledge related to learning outcomes of the topics. During the class, three to four cases were discussed on each topic. A summary of the case and learning points related to learning outcomes were provided by the clerkship director at the end of each case.

### MCQ exam

Students of both years sat a multiple choice question exam at the end of the clerkship. Questions from both final MCQ exams were tagged using the 11 topics and whether it was covered by TBL in the second academic year (Table 1). In addition, both final exam questions were standard set by the clerkship director. Bias should be minimal because trained question writers applying principles of assessment were careful to link questions to course/topic outcomes at the appropriate student level. This process contributed to considerations of differences in difficulty level of questions. Additionally, assessments were multiple choice questions which are graded automatically.

There were 70 questions in the 2015/2016 academic year exam and 100 questions in the 2016/2017 exam. MCQ exams of both years were prepared by the EM clerkship director guided by the course learning outcomes. A committee, which included the EM clerkship director, EM residency program director, and core faculty members of the residency program, completed vetting of the questions. The exam questions were then uploaded into the Assessment Management System of the College and delivered to the students electronically. The total exam time was based on a 90-s answer time for each question, and this applied to both years.

By tagging the questions as TBL versus didactic, we split the questions into two groups. We compared the marks of these two groups while simultaneously adjusting for question difficulty level using standard setting.

### TBL survey

Seeking to achieve a comprehensive understanding of the research question, we sought to converge both quantitative and qualitative data and therefore to triangulate. The experience and personal perspectives of students regarding TBL were of importance in this regard, and a 21-question in-house survey was designed and distributed to the 2016/2017 batch. Nineteen of the questions were based on the 5-point Likert scale. Options were strongly agree (rated 5), agree (4), neutral (3), disagree (2), and strongly disagree (1). Two of the questions were open-ended asking for positive and negative feedback regarding TBL. The anonymous survey was applied via an online application (Socrative), and informed consent was taken from the students at the beginning of the survey assuring freedom to participate and anonymity. Student comments were analyzed to identify strengths, weaknesses, and emerging repetitive themes that give insight into the acceptance and impact of this novel learning modality on students.

### Data collection and analysis

Final exam results were extracted from the College’s Assessment Management System with the following fields: exam year, student ID, question number, topic, covered by TBL, Angoff standard setting cutoff score, and whether the student got that question correct. The survey results were downloaded from the online survey application by question with no student identifying information. Both datasets were imported into Statistical Package for the Social Sciences (IBM-SPSS version 24.0, Chicago, Il, USA) for analysis. Analyses included *t* test, paired *t* test, and ANOVA repeated measures. An alpha level of 0.05 was considered significant.

## Results

### MCQ exam

Table 2 shows a summary of the results for the academic year exams by questions which did not have TBL topics and questions which had TBL topics in the second academic year.

**Table 2:** Summary of the exam results by academic year and TBL topic questions

	TBL not Applied		TBL applied		Difference	*p* value
	2015/2016		2016/2017			
Team-based learning topics	No	Yes*	No	Yes		
Average student mark (%)	70.8	65.4	75.1	70.2		
SD of student mark	0.455	0.476	0.43	0.458		
*N* (student-questions)	2844	2686	3366	3234		
Angoff (cutoff score out of 100)	64.2	66.0	62.9	63.2		
Average overall (6-year) program mark (%)	82.2		83.3		1.14	0.086

*Labelled as TBL topic but not taught using TBL this year, rather applied in the second academic year

The average mark for topics taught via TBL in the second year (71.4%) was significantly higher than the average mark on the same topics taught didactically in the first year (63.9%). There was a significant difference of 7.5% (*t* test, *p* < 0.001). This was adjusted by the Angoff standard setting but not by the overall program mark of the student because the overall program mark difference was not significant (*t* test, *p* = 0.086) (Table 2).

The average mark for topics taught by the didactic/case discussion method in the second year (75.5%) was also significantly higher than the average mark on the same topics taught didactically in the first year (70.3%). A significant difference of 5.2% (*t* test, *p* < 0.001) was recorded, also adjusted by the Angoff standard setting but not by the overall program mark.

The average mark for topics taught via TBL in the second year (70.0%) was significantly lower than the average mark for topics taught by the didactic/case discussion method in the same year (75.2%). A significant difference of 5.2% (*t* test, *p* < 0.001) was recorded. However, the average mark for topics taught didactically in the first year (64.5%) (but labelled as TBL in the second year) was also significantly lower than the average mark for topics taught didactically in the same year (71.6%). There was a significant difference of 7.1% (*t* test, *p* < 0.001).

In the second year, both the TBL and didactic/case discussion topics showed improvement in marks compared to previous year topics which were given via didactic/case discussion. However, TBL topic marks’ improvement was higher than the didactic/case discussion topic marks by 2.3% difference (ANOVA repeated measures, *p* = 0.042, *n* = 144).

Comparing EM questions in the medical exit examination which occurred 2–6 months after the end of the EM clerkship depending on the rotation, we found that there was a significant difference in the questions taught using TBL vs didactic/case discussion for the second year (paired *t* test, *p* = 0.007, *n* = 66). We also examined first year questions (where students were all taught using didactic/case discussion) but compared the TBL-labeled questions (taught in second year as TBL) with the non-TBL-labeled questions and found no comparative significant difference (paired *t* test, *p* = 0.742, *n* = 78).

### TBL survey

A total of 65 out of 66 students completed the survey. This represents a 98.5% response rate. The mean response to questions ranged from 4.03 to 4.63 on a Likert scale of 1 (strongly disagree) to 5 (strongly agree). This indicates that participants tended to strongly agree with the positive statements about TBL (Table 3). Highly rated items were “topic selection was good” (4.63 ± 0.698); “team members encouraged one another to express their opinions” (4.62 ± 0.604); “TBL is a more enjoyable” (4.62 ± 0.823) and “productive method of learning than standard didactic lectures” (4.48 ± 0.903). The lowest mean rated items were “team members used feedback about individual or team performance to help the team to be more effective” (4.03 ± 1.045) and “I prefer all topics in TBL” (4.20 ± 1.107). No statement was rated below 4.03, indicating that students on the whole agreed with the statements.

**Table 3:** EM TBL student survey results for 2016/2017 academic year

Survey question	Average response*	Standard deviation
1. Team members encouraged one another to express their opinions	4.62	0.604
2. My team actively discussed multiple points of view before deciding on a final answer	4.58	0.682
3. Discussions in the team helped me to understand better and organized my knowledge	4.46	0.709
4. Team members used performance feedback to help the team to be more effective	4.03	1.045
5. Team members made an effort to participate in discussion	4.40	0.787
6. Team members more engaged with the topic in TBL than standard didactic lectures	4.38	0.764
7. Team members shared and received criticism without making it personal	4.49	0.616
8. Different points of view were respected by team members	4.62	0.578
9. Team members consistently paid attention during group discussion	4.31	0.900
10. Team members were prepared with the sessions learning outcomes	4.38	0.678
11. Topic selection was good	4.63	0.698
12. Topic learning outcomes covered the entire topic	4.32	0.886
13. Reading material provided covered the learning outcomes	4.40	0.766
14. Time provided for topic preparation was good	4.25	0.902
15. Question for individual and team were directly related to the learning outcomes	4.45	0.708
16. I found TBL more productive method than standard didactic lectures	4.48	0.903
17. I found TBL more enjoyable method than standard didactic lectures	4.62	0.823
18. I prefer all topics in TBL format	4.20	1.107
19. Overall, TBL is a good learning method	4.62	0.654

*65 of 66 students completed the survey. Likert scale was 1 strongly disagree to 5 strongly agree

There were two open-ended questions in the questionnaire which were “Your opinion regarding the Positives or Best Features of TBL” and “Your opinion regarding the Negatives or Worst Features of TBL.” We categorized the responses into four themes.

*Theme 1*: TBL motivates improvement in learning attitudes and approaches

Encourages discussion and peer learning

The best features was the team based learning and discussion among colleagues about the questions during and after TBL session

Team discussion motivates you to study

Each member can explain their opinion and learn from other members’ mistakes, it is a chance for everyone to justify their answers

Keeps me focused… gives you the opportunity to share opinions, facilitates good discussion.

Forces students to remain focused and give it their best, unlike other teaching formats

Self-learning and solving questions have better outcomes compared to usual lecturing

Thank you for the wonderful opportunity to learn through TBL in this rotation, it was the most fun at the same time beneficial way of learning

*Theme 2*: TBL enhances metacognitive growth

Shows you how others think

Tells you how you can improve yourself regarding the selection of answers or thinking process

It gives you the opportunity to really work things out in your mind and correct and reinforce your knowledge

Makes us brain storm and think better, prepares students for international exams

*Theme 3*: Learning together for self and team improvement

As a group we can have different opinions and information which can improve our knowledge rather than a single person, also the team can explain something you were wrong about and it will stick in my head better than reading it

*Theme 4*: Assessment modifications

Some questions have small details which I don’t think it appropriate for our level

Questions can be sometimes confusing especially when the option provided negative scenarios

## Discussion

Consistent with our hypothesis, the average mark for topics taught via TBL in the second year was significantly higher than the average mark on the same topics taught didactically in the first year. We surmise that the two groups of students are equal in every way except by the modality of teaching which is TBL versus didactic/case discussion. They have experienced the same MD program and are only 1 year apart. Their average overall mark in the program is not significantly different. Students in the TBL group enjoyed this strategy and preferred it to didactic lectures.

TBL is designed to reinforce biomedical and clinical concepts, enhance critical thinking, and aid application [15], and therefore, it is unsurprising that topics taught via TBL achieved improved assessment outcomes in end clerkship and final exit examinations 2 to 6 months after the intervention. As in our series, other studies have found that course content learned through TBL enhanced mastery, retention, students’ knowledge-based performance, and enjoyment of the learning process [6, 15]. Improved student performance was also sustained over 8 years in both internal medicine and psychiatry clerkships following inception of TBL compared to delivery of teacher-centered clerkship content [16].

The majority of students expressed satisfaction with TBL, and content analysis of open-ended questions revealed four themes: (i) TBL motivates improvement in learning attitudes and approaches, (ii) TBL enhances metacognitive growth, (iii) learning together for self and team performance improvement, and (iv) needed assessment modifications. Similar themes have emerged in studies evaluating TBL [6, 17, 18].

The average mark for topics taught by didactic/case discussion method in the second year was also significantly higher than the average mark on the same topics taught didactically in the first year. There can be two reasons for this result. One is that second year students’ overall performance is better than first year students, but we already established that there is no significant difference in their overall performance (*p* = 0.086, Table 2). The second is that this is due to the students collaborating more in the second year due to experience in the TBL topics which has carried over to the didactic/case discussion topics, thus supporting a tentative hypothesis that student learning is improved by the TBL method even in topics covered by didactic sessions.

TBL as a variation of flipped classroom modalities [19] aims to reverse traditional lecture and homework processes in a course. During the class time, small group activities are applied with simple delivery of necessary information while students excel their knowledge by reading and watching from provided or online resources [20]. Although time needed to remodel course material is a main challenge for educators of flipped classroom [21], it increases teacher-student interaction time in addition to students’ reports of more engagement and enjoyment [22, 23].

Short- and long-term gains in knowledge following TBL have been demonstrated in pediatric clerkships [15, 24]. Since TBL relies on student pre-session preparation of instructor-assigned materials and on instructor-crafted application exercises in active learning sessions, it is possible that students are not only mastering content through this process of shared student-teacher responsibilities to achieve desire learning outcomes but also applying habits of so called “master learners” more generally, such as self-regulated, reflective, and collaborative learning which aids retention [5]. Our findings are likely multifaceted and more remains to be learned about how long this potential TBL retention effect lasts across diverse settings and content areas [25]. It was demonstrated that improved knowledge retention was achieved via TBL over didactic learning in neurological localization and emergencies for up to 48 h [26]. Additionally, an earlier study using modified TBL for delivery of gross anatomy and embryology showed that knowledge was retained post TBL for up to 19 weeks [27].

The average mark for topics taught via TBL in the second year of our study was significantly lower than the average mark for topics taught didactically in the same year. However, the average mark for topics taught didactically in the first year is also significantly lower than the average mark for topics taught didactically in the same year. We believe that this effect is due to the fact that the topics chosen for TBL were more difficult than those chosen for the didactic teaching. This explains why, in both years, there is a significant reduction in marks when comparing these two groups of topics. Another potential issue in play is the positive effect of assessment in learning [28].

Main drivers for implementation of blended pedagogies, especially TBL, in the EM clerkship were potential to improve knowledge, self-reflection, self-directed, lifelong learning skills, and team-based competencies exemplified by this clerkship. Additionally, in a program with growing student enrollment, the main aim of the clerkship director is to optimize active learning and feedback around clinical problems. This was considered to be best achieved through TBL. TBL is regarded as effective in optimizing student engagement while being less demanding of low faculty-student ratios.

### Limitations

In this study, we were able to compare assessment performance following implementation of didactic/case discussion and TBL learning modalities among non-concurrent cohorts of sixth year medical students. We acknowledge the single institution, two-cohort design, customized format of didactic/case discussion, and TBL teaching modalities. Consideration of potential bias and confounding variables also necessitate acknowledgement. It is possible our didactic/case discussions did not conform to a traditional, purely didactic approach. Similarly, assigning didactic material to learners before class time while using face-to-face time for didactic learning could, strictly speaking, be considered a variant of a flipped classroom approach. TBL is considered a model for flipping the classroom [29].

These learning experiences collectively build learner confidence, self-efficacy, and engagement and exemplify a growing tendency of medical educators to use multiple pedagogies simultaneously, making it more difficult to isolate the independent, unadulterated effects of each. Parmelee et al. also reported that applications of TBL are modified extensively in the literature [30]. There are seven core design elements of TBL, and these elements differ in TBL studies depending on their needs [25]. Our application covers all elements of TBL, except peer assessment following TBL sessions. Although our results significantly favor TBL, small modifications as described in the methods might have hindered more explicit TBL effects in our study.

It is conceivable that difficulty level of questions in both years is not exactly equal. However, question writers have been trained in college-wide faculty development workshops to pay attention to question level when writing questions. In addition, we have standard set the questions and adjusted the marks based on the standard setting to help reduce this bias.

A limitation may be that it is not possible to carry out didactic and TBL sessions with blinded lecturers. This may affect the lecturer efforts during the two different types of sessions which may create some bias. Informal lecturer feedback indicates that the same level of teaching performance was achieved in both types of sessions. However, this was not formally measured.

Additionally, when comparing the student groups in the 2 years, we acknowledge the risk that they may not be exactly the same in some characteristics although we feel that they are similar enough to be compared with low risk of confounders affecting the results. Despite these limitations, we believe that our findings are representative of TBL-exposed and non-exposed cohorts and might be helpful to other medical schools piloting introduction of TBL in clinical clerkships.

Finally, there was no specific question or statement solely about didactic topics in the survey. However, there were several questions aiming to explore student experience comparing TBL and didactic topics. The results were favoring TBL in those questions.

## Conclusions

TBL as part of a blended learning environment facilitated improved knowledge-based performance in an emergency medicine clerkship in our setting following end clerkship and medical school exit assessments, suggesting TBL stimulates long-term retention. This study contributes to the growing body of evidence suggesting the effectiveness of TBL in achieving improved academic performance in the clinical years. While further research is needed to determine the extent of isolated educational effects when using TBL, this study provides more support for its use. The high acceptance of TBL among our students suggests a preference of this learning modality to didactic teaching.

## Abbreviations

CMHS: College of Medicine and Health Sciences
ED: Emergency department
EM: Emergency medicine
PBL: Problem-based learning
TBL: Team-based learning
